# Dietary Restriction and Fasting Arrest B and T Cell Development and Increase Mature B and T Cell Numbers in Bone Marrow

**DOI:** 10.1371/journal.pone.0087772

**Published:** 2014-02-04

**Authors:** Shushimita Shushimita, Marjolein J. W. de Bruijn, Ron W. F. de Bruin, Jan N. M. IJzermans, Rudi W. Hendriks, Frank J. M. F. Dor

**Affiliations:** 1 Department of Surgery, Division of Transplant Surgery, Erasmus MC-University Medical Center, Rotterdam, The Netherlands; 2 Department of Pulmonary Medicine, Erasmus MC-University Medical Center, Rotterdam, The Netherlands; University of Nebraska Medical center, United States of America

## Abstract

Dietary restriction (DR) delays ageing and extends life span. Both long- and short-term DR, as well as short-term fasting provide robust protection against many “neuronal and surgery related damaging phenomena” such as Parkinson’s disease and ischemia-reperfusion injury. The exact mechanism behind this phenomenon has not yet been elucidated. Its anti-inflammatory actions prompted us to thoroughly investigate the consequences of DR and fasting on B and T cell compartments in primary and secondary lymphoid organs of male C57Bl/6 mice. In BM we found that DR and fasting cause a decrease in the total B cell population and arrest early B cell development, while increasing the number of recirculating mature B cells. In the fasting group, a significant reduction in peripheral B cell counts was observed in both spleen and mesenteric lymph nodes (mLN). Thymopoiesis was arrested significantly at double negative DN2 stage due to fasting, whereas DR resulted in a partial arrest of thymocyte development at the DN4 stage. Mature CD3^+^ T cell populations were increased in BM and decreased in both spleen and mLN. Thus, DR arrests B cell development in the BM but increases the number of recirculating mature B cells. DR also arrests maturation of T cells in thymus, resulting in depletion of mature T cells from spleen and mLN while recruiting them to the BM. The functional relevance in relation to protection against organ damage needs to be determined.

## Introduction

Dietary restriction (DR), a moderate reduction in daily calorie intake (20–40% reduction) without causing malnutrition, has been known as an intervention that plays a key role in extending life-span [1], delaying ageing [2] and also in many ageing-related diseases such as diabetes, atherosclerosis, cardiovascular disorders, kidney disease, autoimmune disease and neuronal loss associated with Parkinson’s and Alzheimer’s disease [3]. Both long-term (dietary intervention for more than six months) and short-term (maximum of four weeks) DR, have shown to be beneficial in predicting long-term health and in reducing the rate of cardiovascular disease and insulin sensitivity [4]. Long-term DR has not only proven to be effective in mice [5] but also in various other species like rats [6], flies [7], worms [8], yeast [9], [10], fish [11], non-human primates [12], [13], and in humans [14], [15]. Short-term fasting, another type of DR, has also proven to be beneficial in promoting stress resistance as well as longevity in model organisms and in delaying the growth of cancer cells [16]. Prevention of many ageing-related diseases by DR and fasting has been linked to immunology. Many of the beneficial effects of DR on ageing-related diseases have been attributed to its anti-inflammatory qualities [17]. DR extends life span not only by reducing reactive oxygen species but also by delaying age-related immune deficiencies, such as slowing down thymic involution and declining the production of lymphocytes [18].

No recent data have explicitly shown the effect of DR on the immune system in a broad perspective, but we have demonstrated that short-term DR and fasting have a robust protective effect on ischemia-reperfusion injury (IRI) of both kidney and liver in mice. IRI has been known to be one of the most important inevitable consequences of solid organ transplantation and has a negative impact on both short- and long-term graft survival leading to acute organ failure. Following renal and hepatic IRI, the production of pro-inflammatory cytokines and the subsequent infiltration of the organs by lymphocytes that follows IRI was significantly blunted [19]. Collectively these data strongly imply that the immune system is an important factor in the protective features of DR and fasting. Therefore, we set out to investigate the impact of dietary interventions on the immune system in the same mouse model (10–12 weeks old), but in the absence of IRI. We thoroughly investigated the consequences of DR and fasting on B and T cell development in bone marrow (BM) and thymus respectively. We also studied B cell differentiation in the spleen and mesenteric lymph nodes (mLN) and T cell subtypes in BM and secondary lymphoid organs after DR and fasting.

## Materials and Methods

### Animals

Ten to 12 weeks old C57Bl/6 male mice, weighing 20–25 g, were purchased from Harlan, Horst, the Netherlands. The mice were kept under normal laboratory physiological conditions (temperature 20–24°C, relative humidity 50–60%, 12 hr light/12 hr dark) with 3–4 animals per cage having free access to food (Hope Farms, Woerden, the Netherlands) and water until the start of experimental procedures. All the experimental procedures were performed after approval by the university animal experiments committee (Dutch Ethical Committee, Protocol no. 105-12-12) under the Dutch National Experiments on Animals Act, compiled with Directive 86/609/EC (1986) of the Council of Europe.

### Dietary Regimen

Mice were randomly divided into three groups (n = 8/group): *ad libitum* (AL), two weeks 30% DR (DR) and three days water-only fasting (FA). The first group of AL mice was allowed free access to food and water. In the second group with 30% dietary restriction (4 animals/cage) food intake was weighed daily during the first week (normal consumption is approximately 9.48 g per day) and 30% DR was performed by providing 70% of the food determined by the intake of the previous week. The food was provided at the same time at the end of the day to avoid any discrepancies. The mice in the three-day fasting group were transferred into a new clean cage (to restrain them from eating their own faeces) with free access to water but no food. The fasting regimen also started at the end of the day just before the beginning of the active period of the animals.

### Cell Isolation and Staining

After dietary interventions, mice were exsanguinated by cardiac puncture and thymus, spleen, mLN and bones (bone marrow) were harvested and processed to make cell suspensions. Thymus, spleen and mLN were mashed and passed through 100 µm Nylon cell strainers (BD Falcon™^,^ BD Biosciences Europe, Erembodegem, Belgium). The cell suspensions were made in RPMI-1640 medium (Lonza Europe B.V., Verviers, Belgium) supplemented with 10% FCS (Lonza Europe B.V., Verviers, Belgium) and 1% penicillin/streptomycin (Invitrogen™/Gibco®, Bleiswijk, the Netherlands). To make BM cell suspensions, femur and tibia were crushed using a mortar and pestle followed by re-suspending in RPMI-1640 medium. The total number of live cells were then counted using a Casy TT counter and analyzer (Innovatis, Roche Diagnostics Nederland B.V., Almere, Netherlands) and prepared for flow cytometric analyses.

### Flow Cytometric Analyses

For all lymphoid organs, 2×10^6^ cells were plated in a 96-well plate for each staining. The plate was then centrifuged at 1360 rpm for 3 min. After discarding the supernatant, cells were stained with appropriate mixture of antibodies for staining of B and T cell populations and incubated for 20 min at 4°C. The cells were then washed with 200 µl of FACS buffer and treated with a secondary staining wherever required. After staining the cells with antibodies as mentioned in Table 1, data were acquired on a FACS LSRII™ flow cytometer (BD biosciences,) and analysed using FlowJo™ (Tree Star, Ashland, OR, USA) research software. Dapi and aqua live-dead staining were used to gate on the live cells. These live events were then recorded for the lymphocytes based on forward and side scatter.

**Table 1:** Shows the list of different antibody combinations according to the cell development stages in the various lymphoid organs.

Cell Development	Developmental stage	Antibody combination
B cell development (BM)	Total B cells	CD19^+^B220^+^
	Pro-B cells	CD43^+^CD2^−^IgM^−^IgD^−^
	Pre-B cells	CD43^−^CD2^+^IgM^−^IgD^−^
	Immature B cells	IgM^+^IgD^low^
	Recirculating mature B cells	IgM^+/low^IgD^high^
B cell lymphoid population (Spleen)	Total B cells	CD19^+^B220^+^
	Pro-pre B cells	IgM^low^IgD^low^
	Immature B cells	IgM^+^IgD^−^
	Mature B cells	IgM^+^IgD^+^
	Marginal zone B cells	CD21^+^CD23^−^
	Follicular zone B cells	CD21^−^CD23^+^
T cell development (Thymus)	Double Negative stages (DN)	CD3^−^CD4^−^CD8^−^
	DN1	CD44^+^CD25^−^
	DN2	CD44^+^CD25^+^
	DN3	CD44^−^CD25^+^
	DN4	CD44^−^CD25^−^
	Immature Single Positive (ISP)	CD8^+^CD3^−^CD69^−^
	Double Positive (DP)	CD3^−/low^CD4^+^CD8^+^
	CD8^+^ Single Positive (SP)	CD3^+^CD8^+^CD4^−^
	CD4^+^ Single Positive (SP)	CD3^+^CD4^+^CD8^−^
T cell lymphoid population (Spleen)	Total T cell population	CD3^+^NK1.1^−^
	CD3^+^CD4^+^ SP	CD3^+^CD4^+^CD8^−^
	Memory T cells	CD4^+^CD8^−^CD62L^low^
	Naïve T cells	CD4^+^CD8^−^CD62L^+^CD25^−^
	Naïve Tregs	CD4^+^CD8^−^CD62L^+^CD25^+^

### Chemicals and Reagents Used

Monoclonal antibodies directed against mouse leukocyte populations were obtained from BD Biosciences (BD Biosciences) and eBioscience (Campus Vienna Biocenter 2, Vienna, Austria) unless mentioned otherwise. These monoclonal antibodies labeled with different antibody fluorochrome combinations according to the cell development stages are presented in Table 1.

### Statistical Analysis

Non-parametric paired sample T-tests were performed on the three experimental groups using IBM SPSS Statistics for Windows, Version 20.0 (Armonk, NY: IBM Corp.), while the graphs were plotted using GraphPad Prism version 5.01 for Windows (GraphPad Software, San Diego California USA). P-values ≤0.05 were considered to be statistically significant.

## Results

### Effect of Dietary Restriction and Fasting on Body Weight and Cellularity of Lymphoid Organs

**Figure 1:**
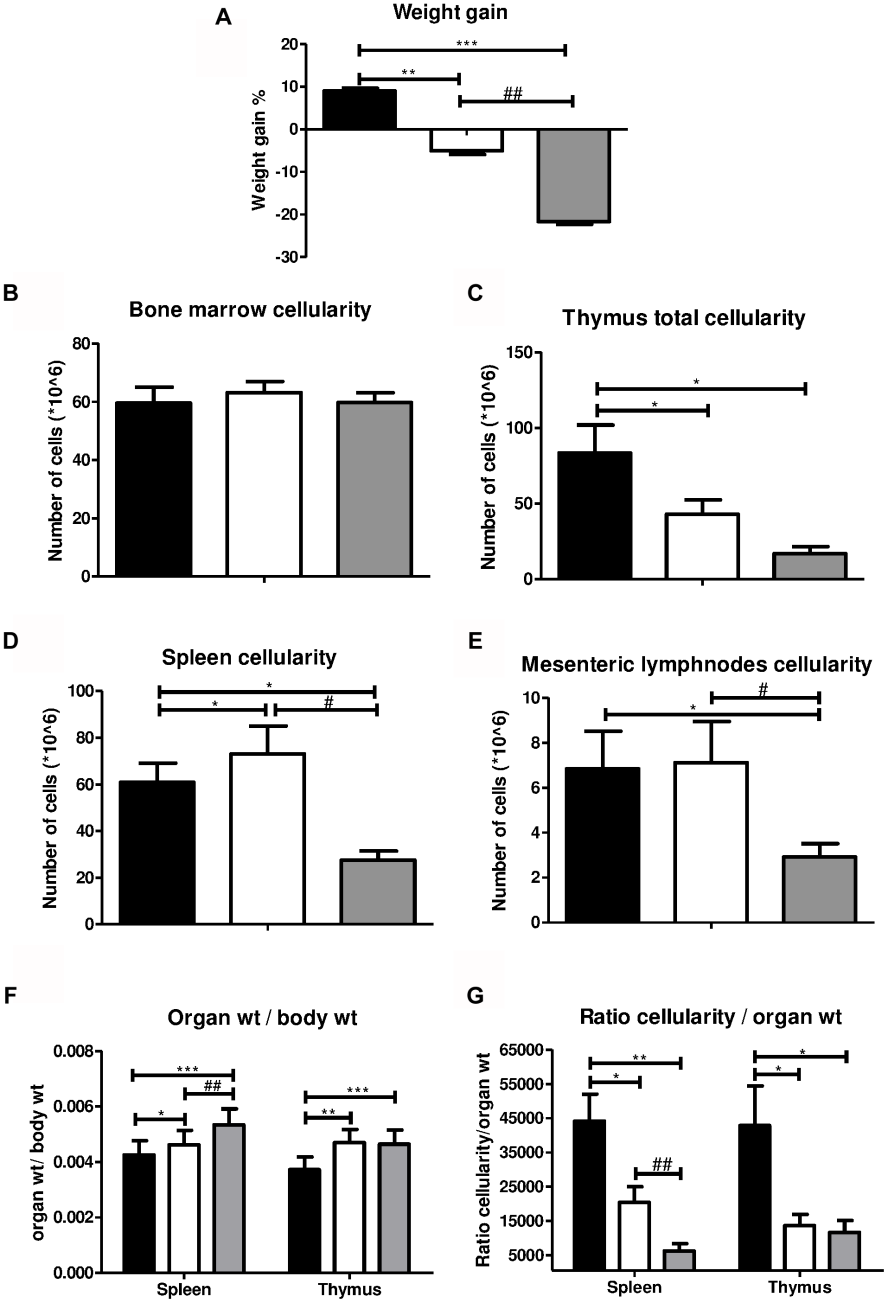
The effect of DR and FA on body weight and cellularity of lymphoid organs. **A)** Both DR and FA cause a significant reduction body weight while **B, C, D**, and **E** show that total cellularity changes take place due to the two dietary interventions. **F)** shows the ratio of spleen and thymus organ weight to the body weight of the mice. **G)** shows the ratio of spleen and thymus cellularity to their respective organ weights. Fig. 1A ** = p<0.001, *** = p<0.0005, ## = p<0.001. Fig. 1C, D, and E *, # = p<0.05. Fig. 1F * = p<0.05, **,## = p<0.005, *** = p<0.0001. Fig. 1G * = p<0.05, **,## = p<0.005. n = 8/group. Ad libitum, 2 weeks 30% DR and 3 days fasting groups are represented by black, white and grey box, respectively. BM –1 hind leg.

In the AL group the body weight increased by an average of 9% (from 24.5+/−1.2 to 26.7+/−0.9 g) (Fig. 1A), while the mean body weight of mice on DR decreased by an average of 6% (from 25.5+/−0.9 to 24.2+/−0.8 g) during the two-week observation period. Three days of fasting reduced weight by approximately 20% (from 26.1+/−2.1 to 20.8+/−0.8 g). Despite the significant weight loss, no mortality or morbidity was observed solely due to the dietary regimens.

The total number of cells in the femur and tibia of the AL mice was 60×10^6^, of the DR group 63×10^6^, and the FA group 60×10^6^ (Fig. 1B). While in the thymus the total number of cells was significantly reduced in the DR group (43×10^6^ cells) and in the FA group (19×10^6^ cells) compared to the AL group (83×10^6^ cells) (Fig. 1C). The total number of cells in the spleen was significantly increased in the DR group (69×10^6^ cells), compared to the AL group (61×10^6^ cells) while it was significantly reduced in FA group (31×10^6^ cells) (Fig. 1D). In mesenteric lymph nodes (mLN), the total cell count in the DR group was not different from the AL group (6.8×10^6^ cells vs. 7.1×10^6^, respectively) while in the FA group, it was found to be significantly decreased (2.9×10^6^ ) compared to both AL and DR groups (Fig. 1E).

The ratio of organ cellularity to the weight of the organ was calculated and was found to be significantly decreased in both the DR and FA group for the spleen as well as the thymus. In the spleen, the ratio in the FA group is also significantly lower than in the DR group. For the thymus, DR and FA ratios are not significantly different. The results of B and T cell numbers and specific subtypes do not always reflect the patterns of the ratios, and thus the proportional reduction of organ weight does not explain all immunological results. An interesting finding is that the organ weight/body weight ratio actually shows an increase for DR and FA, with a significant difference between DR and FA (the relative organ weight in the FA group being higher) (Fig. 1F and G). No reliable weights could be obtained for the mLN, as the number of available mLNs (after dissection from the mesenteric fat) varied per animal.

### Dietary Restriction and Fasting Modulate B cell Development

**Figure 2:**
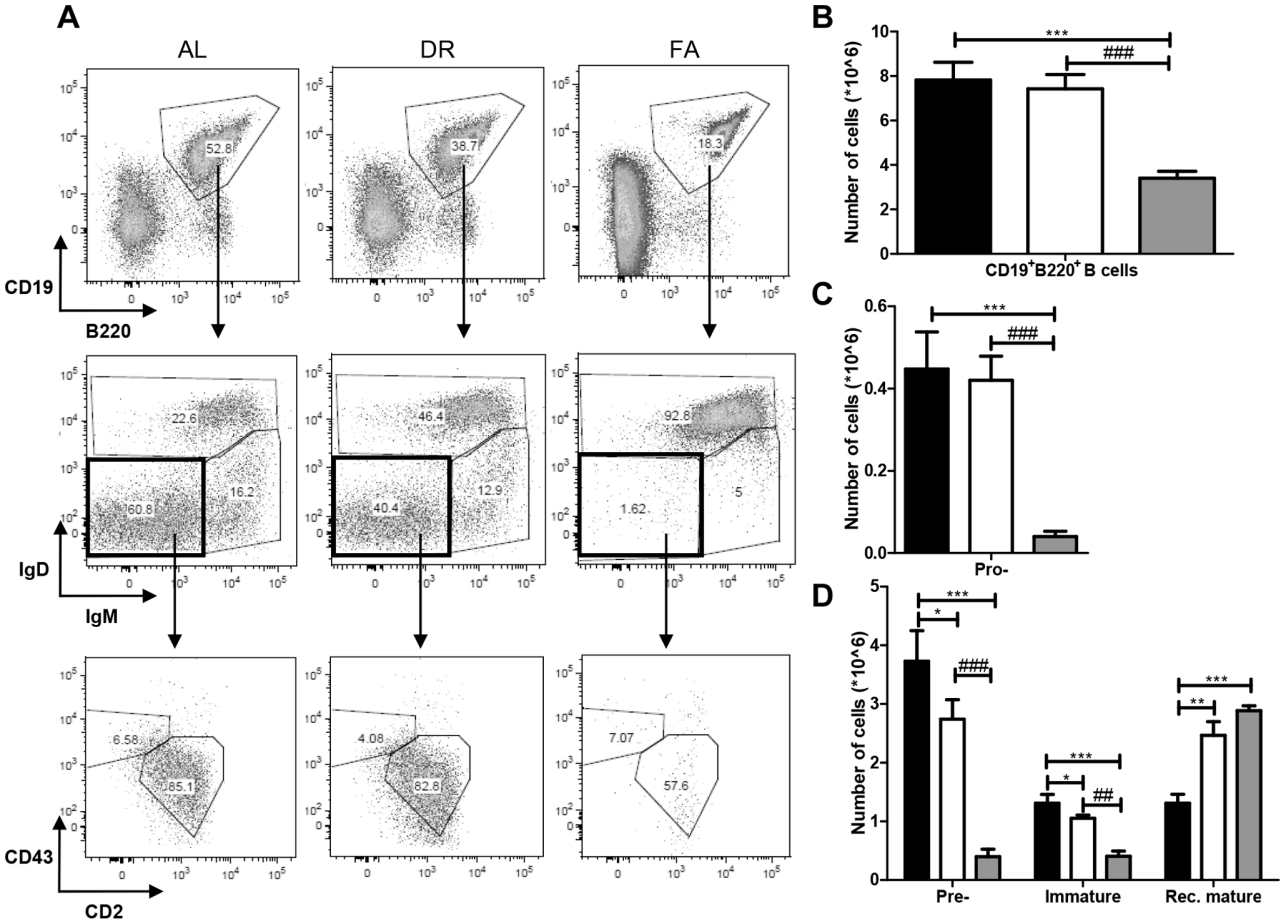
Effects of dietary restriction on B cell development phenotype in the BM. **A)** The first panel of FACS plots represents the plots of CD19 and B220 from the three dietary regimen groups. The second panel shows IgD and IgM populations from CD19^+^B220^+^ B cell fraction with IgM^−^IgD^−^ populations (pro-B+pre-B cells), IgM^+^IgD^low^ (immature B cells) and IgM^+/low^IgD^high^ (recirculating mature) B cells. The third panel represents the CD43^−^CD2 profiles of IgD^−^ IgM^-^ fractions and distinguishes between pro-B (CD43^+^CD2^−^IgM^−^IgD^−^) and pre-B (CD43^−^CD2^+^IgM^−^IgD^−^). **B)** Represents the total B-lineage population (CD19^+^B220^+^) in BM. **C, D)** shows the changes in the B cell development phenotype where both DR and FA cause significant phenotypic alternations in the development stages. Fig. 2B and C *** = p<0.0002, ### = p<0.0004. Fig. 2D *, # = p<0.05, **, ## = p<0.004, *** = p<0.0003. n = 8/group. Ad libitum, 2 weeks 30% DR and 3 days fasting groups are represented by black, white and grey box, respectively.

B-lymphocytes develop in the BM from a common lymphoid progenitor cell, they start differentiating from pro-B cells (CD43^+^CD2^−^IgM^−^IgD^−^) through pre-B cells (CD43^−^CD2^+^IgM^−^IgD^−^), and immature B cells (IgM^+^IgD^low^) to recirculating mature B cells (IgM^+/low^IgD^high^) (Table 1). The total number of CD19^+^B220^+^ B cells found in the AL and DR group was 8×10^6^ while in FA group it was significantly reduced to 3.4×10^6^ (Fig. 2A). The population of surface IgM^-^ B cell precursors in AL mice was found to be 5×10^6^ cells while in DR and FA mice this population was reduced significantly to 3.5×10^6^ and 0.5×10^6^, respectively. Out of the total number of surface IgM^-^ B cell precursors, the number of CD43^+^CD2^−^IgM^−^IgD^−^ pro-B cells was calculated to be 0.5×10^6^ in the AL group and 0.4×10^6^ in the DR group. After FA pro-B cells were significantly reduced to 0.04×10^6^ cells (Fig. 2B). The CD43^+^CD2^+^IgM^−^IgD^−^ pre- B cell population was 4×10^6^ cells in the AL group and was significantly reduced to 2.5×10^6^ cells in the DR group. After FA these numbers were reduced by ten-fold to 0.4×10^6^ cells (Fig. 2C).

We then investigated the phenotypic changes in the immature-, and recirculating mature B cell populations. The total number of immature B cells (IgM^+^IgD^low^) was 1.4×10^6^ in AL mice, 1.1×10^6^ cells in DR, and 0.41×10^6^ cells in FA mice. The recirculating mature B cell (IgM^+/low^IgD^high^) population was 1.5×10^6^ in the AL group while it was significantly increased to 2.7×10^6^ and 3×10^6^ cells in the DR and FA groups respectively (Fig. 2D).

Thus, DR and FA both cause a significant reduction in the pro-B, pre-B and immature-B cell population and a significant increase in recirculating mature B cells in the BM. Compared to DR, FA has a more pronounced effect.

### Dietary Restriction and Fasting Reduce Specialized B Cell Subsets in Secondary Lymphoid Organs

**Figure 3:**
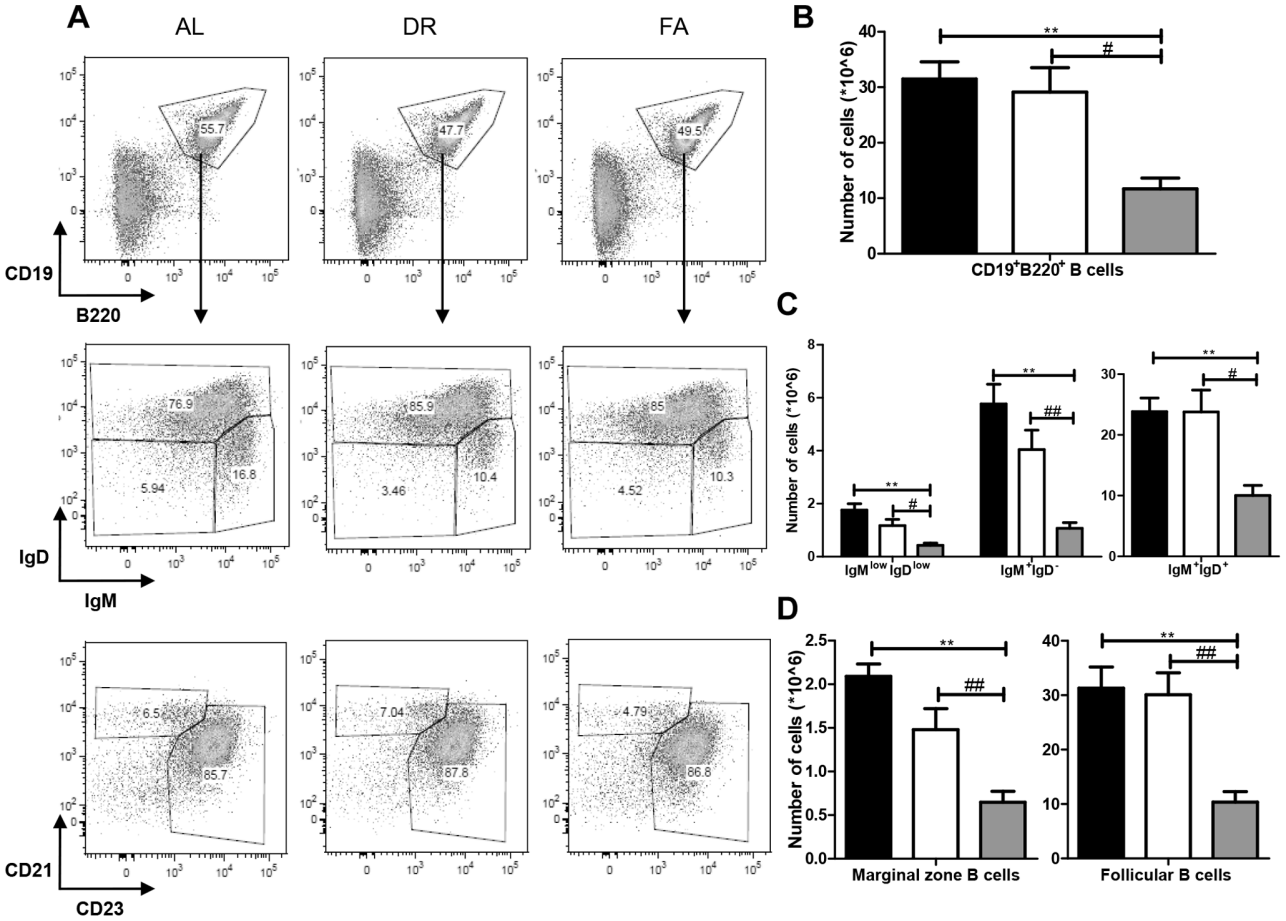
Effects of dietary restriction on the splenic B cell subtypes. **A)** In the first panel, FACS plots of splenic cells plotted against CD19 and B220 in the dietary interventions groups, while the second panel shows the IgM/IgD profiles of gated CD19^+^B220^+^ fractions. The third panel represents the CD21/CD23 profiles of the CD19^+^B220^+^ fractions. **B)** Shows the total CD19^+^B220^+^ B cell cellularity in spleen. **C)** Shows that IgM^low^IgD^low^ was significantly affected by FA but not by DR. A similar trend was also observed with respect to cellularity of immature IgM^+^IgD^−^ and mature IgM^+^IgD^+^ populations. **D)** Represents the cellularity changes in CD21^+^CD23^−^ marginal zone and CD21^−^CD23^+^ follicular B cells. Fig. 3B ** = p<0.001, # = p<0.05. Fig. 3C # = p<0.05 **, ## = p<0.001. Fig. 3D ** = p<0.002, ## = p<0.005. n = 8/group. Ad libitum, 2 weeks 30% DR and 3 days fasting groups are represented by black, white and grey box, respectively.

In the spleen we investigated B cell subtypes such as marginal zone (MZ) and follicular (FO) B cells as well as IgM and IgD low as well as high populations (Table 1). The total CD19^+^B220^+^ B cell population was 32×10^6^ cells in the AL group and 29×10^6^ in the DR group. However, in the FA group the number of B cells was significantly reduced to 11×10^6^ (Fig. 3B). The population of IgM^low^IgD^low^ cells out of the total fraction of CD19^+^B220^+^ B cells in the AL group was 1.8×10^6^ and 1.2×10^6^ in the DR group. In the FA group it was significantly reduced to 0.4×10^6^ cells. The number of CD19^+^B220^+^IgM^+^IgD^−^ immature B cells in the AL group was 5.8×10^6^, whereas the count in the DR group was 4×10^6^. In the FA group the population was significantly reduced to 1.2×10^6^ cells. The population of CD19^+^B220^+^IgM^+^IgD^+^ mature B cells in the AL and DR groups was similar in size (∼24×10^6^ cells and 21×10^6^ cells, respectively), whereas this population was reduced significantly to 10×10^6^ cells in the FA group (Fig. 3C). We also investigated the CD19^+^B220^+^CD5^+^ B1 B cell population, which was found to be 1.4×10^6^, 1.3×10^6^ and 1.1×10^6^ cells in AL, DR and FA groups, respectively (data not shown). B cell subtypes such as CD21^+^CD23^−^ MZ and CD21^−^CD23^+^ FO B cells were also measured in the dietary restricted groups and were all found to be decreased after fasting, while no significant changes were observed after DR (Fig. 3D).

Thus, fasting causes a significant reduction in the total CD19^+^B220^+^ B cell population as well as in IgM, IgD, marginal zone (MZ) and follicular B cell sub-populations in the spleen, while DR has no significant effect. However, distribution over mature/immature on MZ/FO is not affected.

Analysis of B cell sub populations in the mLN revealed significant changes in the populations of CD19^+^B220^+^ B cells, which were observed to be 2.5×10^6^, 2×10^6^ and 0.6×10^6^ in AL, DR and FA, respectively (Fig.S1).

### Thymic T cell Development is Arrested by Dietary Restriction and Fasting

**Figure 4:**
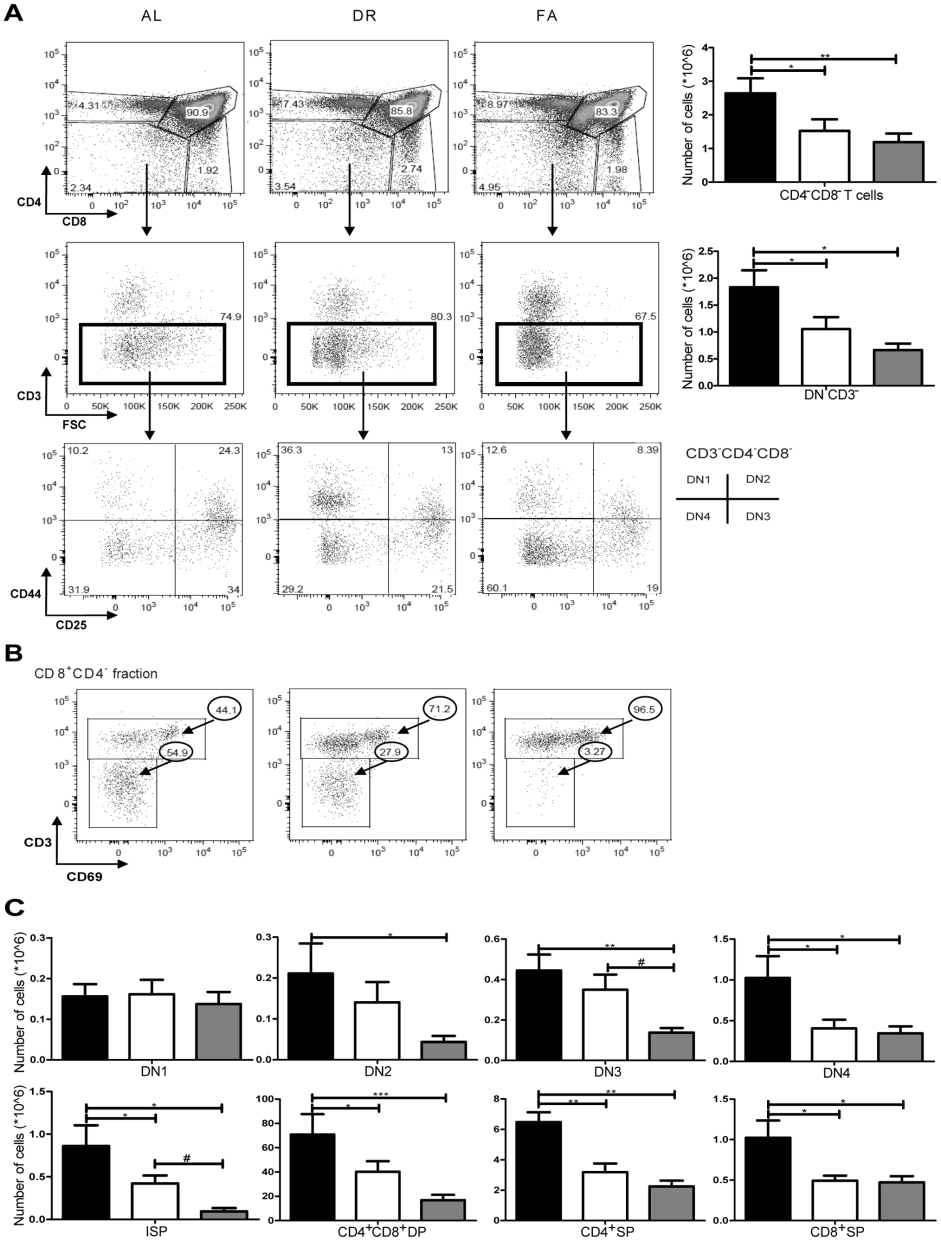
Effects of dietary restriction on T cell development phenotype in thymus A) FACS plot of CD4 against CD8 in the first panel and the corresponding CD4^−^CD8^−^ cellularity graph. The second panel represents the CD3/FSC FACS profiles of CD4^−^CD8^−^ DN fractions along with the corresponding cellularity graph of DN CD3^−^. The third panel of plots shows CD44/CD25 profiles of DN CD3^−^ fraction. This panel shows the DN1-DN4 stages in the dietary intervention groups. B) FACS plots show the expression of CD3/CD69 profiles of CD8^+^CD4^−^ fractions representing the CD3^−^CD69^−^ISP and CD3^+^CD8^+^ SP cells C) The upper and lower panel of graphs show the phenotypic changes taking place during the development stages starting from DN stages to CD3^−^CD69^−^ ISP, CD4^+^CD8^+^ DP and CD4^+^ SP and CD8^+^ SP. Fig. 4A and C *, # = p<0.05, ** = p<0.001, *** = p<0.007. n = 8/group. Ad libitum, 2 weeks 30% DR and 3 days fasting groups are represented by black, white and grey box, respectively.

T cell development (thymopoiesis) and differentiation occur in discrete stages starting with double negative stages (DN) in the thymus. In mice, it has been reported that there are four DN stages distinguished by the expression of CD25 and CD44 surface markers (Table 1): CD44^+^CD25^−^ for DN1, CD44^+^CD25^+^ for DN2, CD44^−^CD25^+^ for DN3 and CD44^−^CD25^−^ for DN4 phenotypic stages. When we gated the CD4^−^CD8^−^ total DN population, we found a decreased number in both DR and FA groups (1.5×10^6^, 1×10^6^ respectively) compared with 2.6×10^6^ in the AL group. We observed the same trend when we gated the total DN CD3^−^ T cell population, which was found to be decreased in DR and FA groups from 1.8×10^6^ in AL to 1×10^6^ and 0.7×10^6^, respectively. We studied the DN stages from this DN CD3^−^ T cell population. No significant changes were observed in the population of DN1 stage while the other DN stages were found to be decreased due to both DR and FA (Figure 4A, 4C). The size of the DN2 population in the AL group was 0.2×10^6^ while this was 0.14×10^6^ in the DR group and 0.04×10^6^ in FA group. Similarly, the DN3 population in DR and FA was reduced from 0.44×10^6^ cells in the AL group to 0.35×10^6^ cells in the DR group to 0.14×10^6^ cells in the FA group. The DN4 populations also showed a similar decrease with 1×10^6^ cells, 0.41×10^6^ cells and 0.34×10^6^ cells in AL, DR and FA group respectively (Fig. 4C).

After the DN stages, thymocytes which start CD8 upregulation progress to immature single positive (ISP) CD8^+^CD3^−^CD69^−^ thymocytes. The number of CD8^+^CD3^−^CD69^−^ ISP cells in the thymus was 0.86×10^6^, 0.42×10^6^, 0.09×10^6^ in AL, DR and FA groups, respectively, while that of mature CD8 single positive (SP) was 1×10^6^ in the AL group, 0.5×10^6^ in both DR and FA groups (Fig. 4B, 4C).

Once the cells have passed through the ISP stage they progress from double positive (DP, CD4^+^CD8^+^) to single positive (SP) stage (CD4^+^CD8^−^ or CD4^−^CD8^+^). The number of DP cells was 71×10^6^ in the AL group and was significantly decreased in the DR (40×10^6^) and FA groups (17×10^6^). We also found a significant decrease in SP CD4^+^ T cells from 7×10^6^ in the AL group to 3.2×10^6^ in the DR group and to 2.2×10^6^ in the FA group. Similarly, the SP CD8^+^ T cell population was found to be 2×10^6^, 1×10^6^ and 0.7×10^6^ in AL, DR and FA groups, respectively (Fig. 4C). Thus, DR and FA both cause a significant inhibition of thymocyte development, with severely reduced numbers of thymocyte subpopulations from the DN2 and DN4 stage onwards.

### Fasting Reduces the Number of CD3^+^ NK1.1^−^ T cells and Tregs in the Spleen and Mesenteric Lymph Nodes

**Figure 5:**
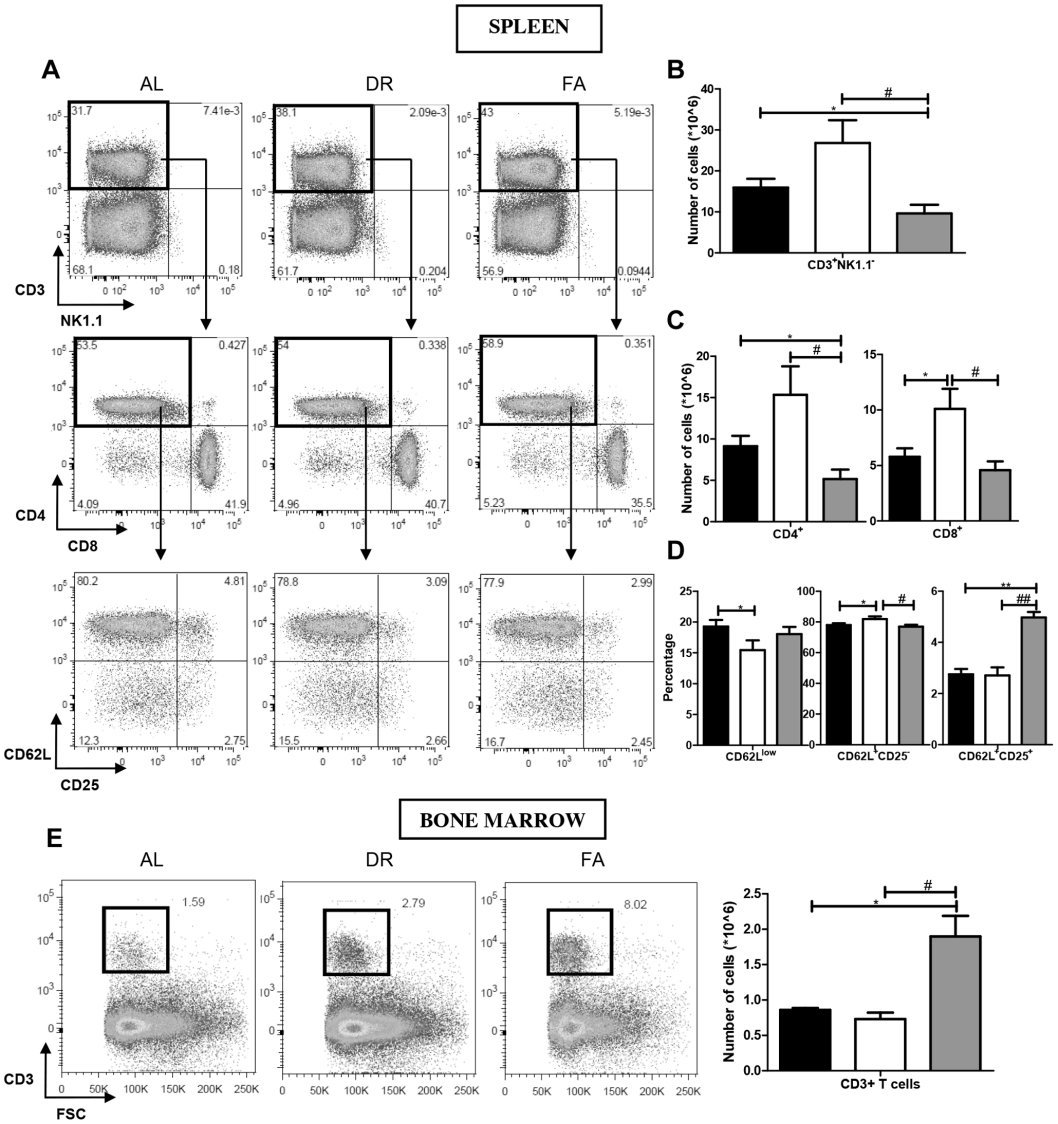
Effects of dietary restriction on spleen and BM T cell lymphoid populations. **A)** The first panel represents the FACS plot of the total CD3^+^NK1.1^−^ T cell population in spleen. The second panel of FACS plots show the CD4/CD8 profiles of the gated total CD3^+^NK1.1^−^ fractions while the third panel of FACS plots represents the CD62L/CD25 profiles of the total CD4^+^CD8^−^ fractions. **B)** The graph represents the total CD3^+^NK1.1^−^ T cell cellularity. **C)** Shows the total number of CD4^+^ and CD8^+^ T cells. **D)** Shows the percentages of CD62L^low^ memory T cells, CD62L^+^CD25^−^ naïve T cells and CD62L^+^CD25^+^ Tregs populations. **E)** Shows the CD3^+^ T cell changes taking place in BM. Fig. 5B * = p<0.01, # = p<0.05. Fig. 5C *** = p<0.0005. Fig. 5D *,# = p<0.05, **,## = p<0.001. Fig. 5E *,# = p<0.02. n = 8/group. Ad libitum, 2 weeks 30% DR and 3 days fasting groups are represented by black, white and grey box, respectively.

We investigated naïve, memory and naïve regulatory T cells (Tregs) which are known to be distinct cell populations residing in the spleen. We first gated on the true CD3^+^ NK1.1^−^ T cell (to distinguish CD3^+^ T cells from NKT^+^ cell population, Fig. 5A). The total CD3^+^NK1.1^−^ T cell population in AL group was 16×10^6^ cells, and 27×10^6^ cells in the DR group. In the FA group there was a significant reduction to10×10^6^ cells (Fig. 5B). Out of the total CD3^+^NK1.1^−^ T cell population we plotted for CD4^+^CD8^−^ T cells (Fig. 5C) and found that there was a significant decrease in the FA group (5×10^6^, respectively) as compared to 9×10^6^ in the AL group, while counts in the DR group remained unchanged. The same could be demonstrated for CD4^−^CD8^+^ T cell population, which was found to be increased in the DR group and decreased in the FA group in comparison to the AL group (6×10^6^, 10×10^6^ and 5×10^6^, respectively). When we plotted CD62L/CD25 profiles from CD4^+^CD8^−^ fractions, we found that the percentages of CD62L^low^ memory T cells were decreased in the DR group, while no significant difference was observed in the FA group. However, with respect to CD62L^+^CD25^−^ naïve T cells, we found an increased percentage in the DR group and a decreased percentage in the FA group, although differences were small. For CD62L^+^CD25^+^ naïve Treg, we found a significantly increased percentage in the FA group (Fig. 5D). The T- to B cell ratio in the spleen was significantly increased in the FA group while no changes were observed in the DR group (Fig.S2). Thus, FA causes a significant reduction in the CD3^+^NK1.1^−^ T cell population together with an increase in the number/percentage of Tregs. T cells in the mLN showed a similar trend as observed in the spleen (Fig.S3).

### Dynamics of T cells in Bone Marrow

Although the total number of CD3^+^ T cells in BM is normally quite low, we found significant changes in both of the dietary intervention groups. The total number of CD3^+^ T cells in BM in the AL group was 0.86×10^6^, which was decreased to 0.73×10^6^ cells in the DR group and significantly increased to 1.9×10^6^ cells in the FA group (Fig. 5E).

## Discussion

Dietary restriction has not only shown to be effective in reducing the major inflammatory disturbances in ageing-related diseases but has also shown to increase stress resistance and suppress organ damage and inflammation following toxic or ischemic insults. We previously investigated the effect of two weeks of 30% DR and three days of fasting on IRI in the kidney and liver and found that the inflammatory reaction was strongly suppressed [19]. To assess the involvement of the immune system in the beneficial effects of DR, we investigated the effect of these dietary restriction regimes on lymphoid and myeloid cell populations in all the major primary and secondary lymphoid organs, in the present study. The regimen of two weeks of 30% DR and three days of fasting causes a decrease in the total CD19^+^B220^+^ B cell population in BM. These dietary interventions also cause depletion of all B cell development stages, and induce an increase in IgM^+^IgD^+^ B cells in the BM, which are B cells that return to the BM after they have completed their maturation process in the periphery [20]. Since we see an increased phenotypic population of B cells in the BM due to both dietary interventions, we hypothesize that this is related to the depletion of the same set of B cells from the spleen and mLN and suggests either recruitment of peripheral B cells to the BM or that the increase may be due to changes in cellular migration or homing. Apparently, dietary interventions are causing BM to save more energy by halting the production of more B cells and concomitantly directing the BM to spare its energy to maintain its metabolic imbalances.

DR and FA both induced a depletion of immature, transitional B cells (CD19^+^B220^+^IgM^+^IgD^−^) as well as mature B cell populations (CD19^+^B220^+^IgM^+^IgD^+^) in the spleen. We have also observed that FA causes significant depletion of marginal zone and follicular B cells. The same holds true for B cell changes taking place in mLN. Hence, all the splenic B cell sub-populations were observed to be significantly reduced in the FA group compared to both AL and DR, demonstrating that FA has a more pronounced effect on the B cell population in the spleen as compared to that of DR. The reason behind FA having a more pronounced effect than DR is not yet clear. We hypothesize that these are two different types of dietary interventions in which the mice are subjected to two weeks of 30% DR and three days of water-only fasting. One explanation may be the difference and duration of the two interventions. The weight loss observed in the DR group is greater during the first few days after which the weight is returned to normal suggesting that they acclimatize and adjust to the amount of food they receive. However, the mice in the FA group constantly lose weight for three days and we have noticed the fact that they also dehydrate themselves by not drinking water**.** We have learned that both these dietary interventions are two different types of stressors leading to pronounced immunological changes due to FA and in a lesser extent due to DR. The exact mechanism behind this phenomenon still merits further investigation, as both interventions lead to robust protection of renal IRI [19]. It is not yet clear where the depleting B cells from spleen and mLN migrate to during the event of dietary interventions but one of the hypotheses is that these cells are (partially) recruited to the BM, as mentioned before. Alternatively these cells may move to the Peyer’s patches, which are a component of the gut-associated lymphoreticular tissues (GALT), and are the major site of antigen uptake to induce mucosal secretory-IgA antibody responses [21].

In the thymus both DR and FA cause major changes in T cell development. Both these dietary interventions cause depletion of the T cells starting at the DN stage and progressing through the ISP and DP stages. Thus, dietary interventions arrest the development and maturation of T cells and hence arrest thymopoiesis. FA also causes an increase in mature T cells in the BM which is an unusual phenomenon, and we are currently investigating the explanation thereof.

We have already mentioned the beneficial effects of dietary interventions not only in ageing-related diseases but also in organ transplantation. Information currently recovered from studies, especially in the field of organ transplantation (IRI), demonstrates that there is an enormous amount of infiltration and activation of the lymphoid cells causing damage to the organ. We find that dietary interventions cause a depletion in pro-, pre-, and immature B cell populations, while they increase the population of recirculating mature B cells in BM which may be a mechanism by which DR and FA cause robust protection. From the various IRI models using mice with defects in T/B cell maturation, we have also learned that these mice are protected against severe effects of IRI. Rabb et al. showed that athymic *nu/nu* mice were protected from acute kidney injury and upon adoptive transfer of T cells into these mice renal injury was reconstituted following IRI. This demonstrated that T cell deficiency conferred protection from acute kidney injury in this strain [22]. Several other groups have also illustrated that specific T cell subtypes for examples, RORγt+ T cells [23], CD4/CD8^−/−^ [24] and γδ T [25] cells play an important role in the pathophysiology of IRI. We are currently investigating the effects of DR and FA on different unconventional T cell subsets and whether these effects may be related to the protection by dietary interventions.

Besides the importance of T cells in IRI, Rabb’s group has shown that B cell deficient mice (µMT (Igh-6^tm1Cgn^) mice) are protected against IRI as well, thereby confirming the role of B cells in IRI [26]. We are not aware of published studies that address different B cell subtypes and their role in IRI. From our studies we conclude that there is a reduction in both B (all subtypes) and T cells in the spleen induced by DR and FA (which may be protective against renal IRI), in accordance with Rabb’s findings.

Tanaka et al. [27] stated that deprivation of nutrition induces atrophy of the thymus and spleen and also reduces the number of circulating B and T cells which is what we observed in our mouse model of DR and FA. The major factors responsible for these changes in B and T lymphocyte population are likely hormones such as corticosterone, for which serum levels are high in DR and significantly higher after FA [28]. Leptin, which has all the characteristics of a pro-inflammatory cytokine, has been shown to link nutritional status with immune responses and has been demonstrated to play a major role in altering the B cell development during FA [27], [29]. A recent study by Fujita et al., also report the role played by leptin in B cell homeostasis by inhibiting apoptosis. They showed that after FA for 60 hours there is an increased rate of B cell apoptosis to ∼14%. However, after leptin administration during the FA period, a proportional reduction in the apoptotic cells was observed. This can most likely be attributed to the stabilization of metabolic and endocrine disturbances induced by FA regimen [30]. Together these data suggest that apoptosis may be a mechanism by which the loss of lymphocytes during DR can be explained.

The mechanism behind life-span extension through DR has been primarily reported to be due to the nutrient-sensing pathways [31]. As reported by these authors, many of the mutations that extend life span decrease activity of nutrient-signaling pathways, including the mammalian target of rapamycin (mTOR) pathways suggesting induction of a physiological state similar to that resulting from periods of food shortage. Other research groups have also highlighted the importance of the mTOR pathway in increasing the life span through DR via treatment with rapamycin which forms an inhibitory complex with TOR kinase and prevents proteins synthesis and cell growth [32], [33]. A recent publication by Yilmaz OH. et al. has demonstrated that DR/CR acts by inhibiting activity of mTOR complex 1, of the intestinal stem cells thereby resulting in promoting a more favourable stem-cell microenvironment [34]. However, no mechanism is known with respect to the effect of DR on B and T cell development. A study by Zhang et al. has shown that mTOR inhibition affects T cell differentiation and function by a decrease in the number of thymocytes and CD4^+^, CD8^+^ T cell populations in spleen. It is also shown that inhibition of mTOR causes alterations in T cell activation, trafficking, or homeostasis and differentiation. mTOR inhibition does not only affect T cells; also B cells are affected by mTOR inhibition. Experiments performed in mTOR inhibition model showed that both the numbers and percentages of B220^+^ B cells have been reduced in the spleen and BM [35], one of the phenomena that we observed in our DR and FA model. This suggests that alterations in B and T cell populations observed due to DR and FA may act through the inhibition of mTOR although a thorough mechanistic study needs to be performed.

Engagement of both the innate and adaptive immunity in IRI has been comprehensively reviewed in the article by Rabb et al. Based on numerous studies highlighted in the article, a robust inflammatory process engages cells and elements of both the innate and adaptive immune responses in causing initial organ injury and mediating long-term structural changes, suggesting a complex role for the immune system [36]. We hypothesize that DR and FA act as a low-level stressor inducing basic protective mechanisms that modulate inflammatory responses to harmful danger signals as well as bringing about alterations in B and T cell development. These dietary interventions also cause recruitment of recirculating mature B cells and CD3^+^ T cells to the BM, possibly contributing to the organ saving more of its energy by not producing more of the B cells and thereby directing the BM to more energy consumption/storage. Hence, both DR and FA cause major alterations in B and T cell development and differentiation in primary and secondary lymphoid organs. The immunological effects observed due to DR are not the same as those observed due to FA, although both DR and FA are protective against the adverse effects of renal IRI. These different immunological outcomes suggest that different mechanisms of action may play a role in the protection against IRI, and that the immunological effects of the dietary interventions may not be crucial in the protective effect nor that these are bystander effects. This clearly merits further investigation.

It was shown by Fernandes et al. in a murine model that DR caused an increase in lifespan, even after infection with murine AIDS [37]. Influenza infection has been known as one of the diseases that has a major impact on elderly human health. Effros et al. demonstrated that DR had a protective effect against influenza infection in aged mice [38]. This implies that the function of the immune system in diet restricted mice is not compromised, and that the changes observed due to dietary interventions in our model with respect to B and T cell development may be associated with improved immune function. There have been several more recent studies highlighting the effect of influenza infection in DR treated mice in which decreased survival in DR mice has been observed [39], [40]. This raises questions about the relevance of the major phenotypic immunological changes taking place in different immune compartments. The functional consequences of these phenotypic changes are currently unknown. In this context, it has been shown by Pahlavani et al., that DR significantly enhances lymphocyte function as assessed by mitogen-induced lymphocyte proliferation, and attenuates age-related decline in immunologic responses such as cytokine production, antibody response to sheep red blood cells and NK cell activity. Consistent with our findings, it has been reported that DR delays a rise in memory T cells during ageing and thus retains a higher number of naive T cells in aged mice and in this way delays immunosenescence [41].

Dietary restriction is the most efficacious and non-invasive method of causing an increase in lifespan of laboratory animals. The decrease in age-associated diseases is directly correlated to the increased longevity due to DR. Only a few studies have shown that DR brings about these protective effects by modulating changes in the immune system by means of delaying immunosenescence and also by stress resistance. However, the mechanism by which DR exerts its protective effect remains unclear. We have shown that two different short-term dietary interventions cause alterations in all lymphoid compartments. When compared to two weeks of 30% DR, three days of short-term fasting has a more pronounced effect. Both DR and FA halt B and T cell development and also cause recruitment of recirculating mature B and T cells to the BM, which has not been observed previously. Whether these major immunological changes observed after dietary interventions are a bystander effect or directly mechanistically related remains to be elucidated. Our laboratory is currently conducting research projects determining the functional consequences of these immunological changes and how these alterations may be involved in the beneficial effects of DR on life-span and acute stress resistance.

## Supporting Information

Figure S1 The effect of DR and FA on CD19^+^B220^+^ B cells in mesenteric lymph nodes. Both FA and DR cause a significant decrease in the total B cell as compared to the AL group. * = p<0.05, ## = p<0.005. n = 6/group. Ad libitum, 2 weeks 30% DR and 3 days fasting groups are represented by black, white and grey box, respectively.
(TIF)

Figure S2 The effect of DR and FA on T/B cell ratio in spleen. FA causes a significant increase in the T/B cell ratio while DR has no effect as compared to the AL group. *, # = p<0.05. n = 8/group. Ad libitum, 2 weeks 30% DR and 3 days fasting groups are represented by black, white and grey box, respectively.
(TIF)

Figure S3 The effect of DR and FA on mesenteric lymph node T cell lymphoid population. DR does not cause any significant change while FA causes a significant reduction in the total CD3^+^NK1.1^−^ T cell population as compared to the AL group. *,# = p<0.05. n = 6/group. Ad libitum, 2 weeks 30% DR and 3 days fasting groups are represented by black, white and grey box, respectively.
(TIF)
