# Supplementary figures and images for: Dietary Restriction and Fasting Arrest B and T Cell Development and Increase Mature B and T Cell Numbers in Bone Marrow
Source: PLoS One. 2014 Feb 4;9(2):e87772. doi: 10.1371/journal.pone.0087772 (PMC3913690; doi:10.1371/journal.pone.0087772)

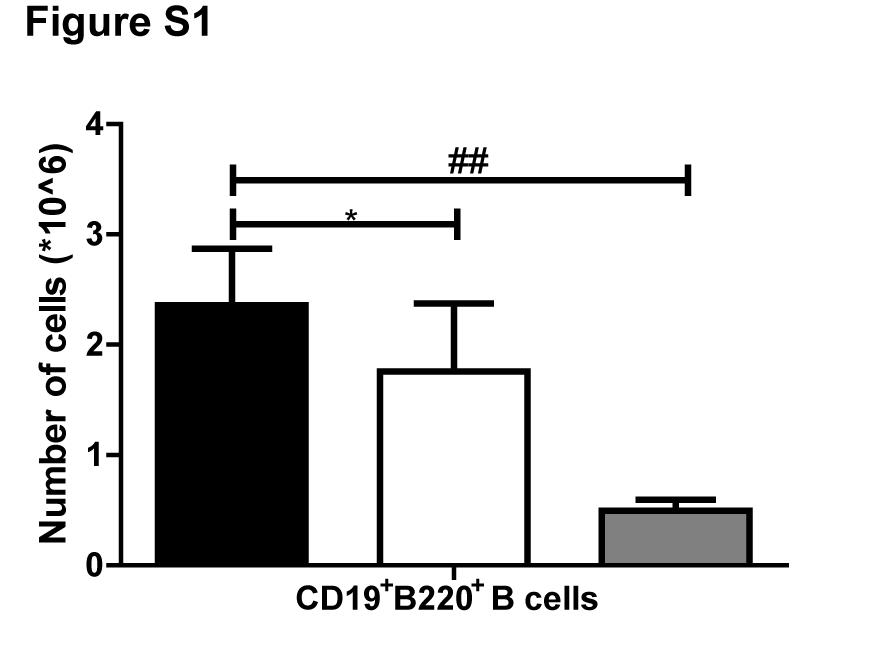

Supplement: Figure S1 — The effect of DR and FA on CD19+B220+ B cells in mesenteric lymph nodes. Both FA and DR cause a significant decrease in the total B cell as compared to the AL group. * = p<0.05, ## = p<0.005. n = 6/group. Ad libitum, 2 weeks 30% DR and 3 days fasting groups are represented by black, white and grey box, respectively. (TIF) [file pone.0087772.s001.tif]

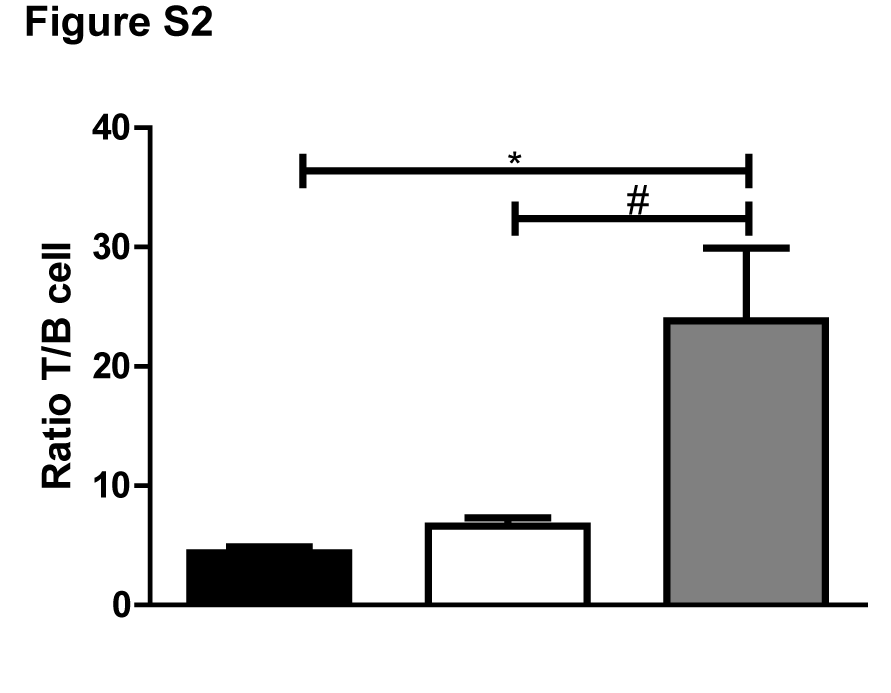

Supplement: Figure S2 — The effect of DR and FA on T/B cell ratio in spleen. FA causes a significant increase in the T/B cell ratio while DR has no effect as compared to the AL group. *, # = p<0.05. n = 8/group. Ad libitum, 2 weeks 30% DR and 3 days fasting groups are represented by black, white and grey box, respectively. (TIF) [file pone.0087772.s002.tif]

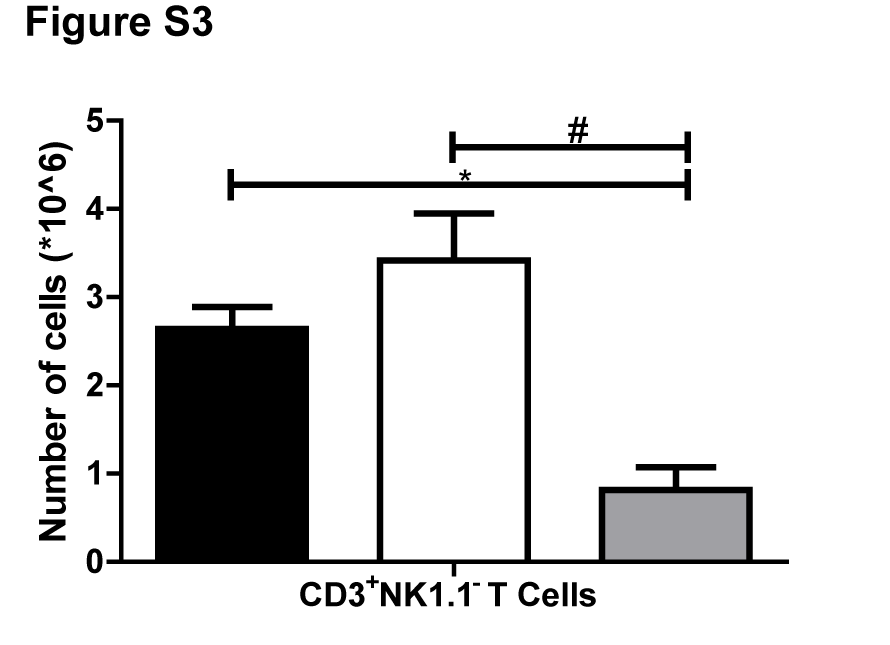

Supplement: Figure S3 — The effect of DR and FA on mesenteric lymph node T cell lymphoid population. DR does not cause any significant change while FA causes a significant reduction in the total CD3+NK1.1− T cell population as compared to the AL group. *,# = p<0.05. n = 6/group. Ad libitum, 2 weeks 30% DR and 3 days fasting groups are represented by black, white and grey box, respectively. (TIF) [file pone.0087772.s003.tif]
